# Epidemiology and Associated Factors of Superimposed Preeclampsia in Pregnant Women With Chronic Hypertension: A Retrospective Multicenter Cohort Study

**DOI:** 10.1155/jp/3799170

**Published:** 2026-03-03

**Authors:** Anya Han-idhikul, Kiattisak Kongwattanakul, Ratana Komwilaisak, Piyamas Saksiriwuttho, Sukanya Chaiyarach, Chatuporn Duangkam, Sathida Chantanavilai, Kaewjai Thepsuthammarat

**Affiliations:** ^1^ Department of Obstetrics and Gynecology, Faculty of Medicine, Khon Kaen University, Khon Kaen, Thailand, kku.ac.th; ^2^ Department of Obstetrics and Gynecology, Khon Kaen Hospital, Khon Kaen, Thailand, kkh.go.th; ^3^ Clinical Epidemiology Unit, Faculty of Medicine, Khon Kaen University, Khon Kaen, Thailand, kku.ac.th

**Keywords:** chronic hypertension, epidemiology, expectant management, neonatal outcome, perinatal outcome, preeclampsia, severe features

## Abstract

**Objectives:**

The objective of the study is To to determine the incidence of superimposed preeclampsia among pregnant women with chronic hypertension and associated factors of superimposed preeclampsia:preeclampsia.

**Material and Methods:**

A retrospective multicenter cohort study was conducted at Srinagarind Hospital, Khon Kaen University, and Khon Kaen hospitalHospital, Thailand, involving women who were admitted between November 1, 2017, to and October 31, 2022. The pregnant women who had been diagnosed with chronic hypertension were identified and their medical records were reviewed for incidence of superimposed preeclampsia. Various characteristics were examined to compare maternal complications, perinatal outcomes, and associated factors. Logisticfactors.Logistic regression analysis was performed to identify factors associated with superimposed preeclampsia.

**Results:**

There was a total of 33,018 deliveries during the study period, out of which 406 (1.2%) women with chronic hypertension were identified. Superimposed preeclampsia occurred in 199 women, accounting for a rate of 49.0% (95% confidence interval; [CI] 44.1–53.9). One hundred and nineteen women (59.8%) were diagnosed with superimposed preeclampsia with severe features, and 80 (40.2%) without severe features. A mean arterial pressure (MAP) ≥100≥ 100 mmHg during pregnancy, the requirement of two or more antihypertensive agents, and a history of previous preeclampsia were significantly associated with an increased risk of superimposed preeclampsia (adjusted odds ratio; [OR] 9.97, 95% CI95%CI 5.95 – 16.71, adjusted OR 2.31, 95% CI95%CI 1.30 – 4.12, and adjusted OR 4.52, 95% CI95%CI 1.86 – 10.98, respectively).

**Conclusion:**

Approximately half of the women with chronic hypertension developed superimposed preeclampsia. MAP ≥ 100 mmHg, the requirement of two or more antihypertensive agents, and a history of previous preeclampsia might be useful tools for predicting superimposed preeclampsia.

## 1. Introduction

Chronic hypertension in pregnancy is defined as hypertension diagnosed before pregnancy or before 20 weeks of gestation and hypertension during pregnancy that does not resolve in the postpartum period (1). Chronic hypertension complicates 1%–5% of pregnancies (1, 2). The rate of chronic hypertension increased, on average, by 6% per year and is expected to continue due to the increasing maternal age and obesity (3, 4). Chronic hypertension may result in significant maternal, fetal, and neonatal morbidity and mortality, including superimposed preeclampsia, cesarean delivery, gestational diabetes, postpartum hemorrhage, fetal growth restriction (FGR), preterm birth, neonatal intensive care unit (NICU) admission, stillbirth, and neonatal death (2, 5, 6).

Superimposed preeclampsia occurs in 20%– to 60% of women with chronic hypertension;, compared with women without chronic hypertension, the risk of preterm superimposed preeclampsia is 5– to 6 times higher (2, 5, 7, 8). In superimposed preeclampsia, compared with chronic hypertension alone, there is a higher risk for adverse outcomes, including cesarean delivery, placental abruption, low birth weight, preterm birth, and NICU admission (9, 10).

The risk for superimposed preeclampsia correlates with the severity of baseline hypertension and the need for antihypertensive treatment. Previous study reported significantly lower adverse pregnancy outcomes in chronic hypertension women with pre‐enrollment blood pressure (BP) less than 140/90 mmHg (7). Traditionally, the criteria for diagnosing hypertension in pregnancy define as systolic blood pressure (SBP) ≥ 140 mmHg, diastolic blood pressure (DBP) ≥90 mmHg, or both (1). Recent criteria for diagnosing hypertension in adults from the American College of Cardiology (ACC) and the American Heart Association (AHA) include classifying BP into four categories; the recommendations suggest the benefit of antihypertensive treatment in nonpregnant adults with Stage 1 hypertension (SBP of 130–139 mmHg or DBP of 80–89 mmHg) for the prevention of cardiovascular disease (CVD) (8). These changes in recommendations may result in alterations to the incidence rates and pregnancy outcomes of superimposed preeclampsia among women with chronic hypertension.

Elevated mean arterial pressure (MAP) reflects increased systemic vascular resistance and endothelial dysfunction, leading to impaired uteroplacental perfusion, an important mechanism in preeclampsia. Similarly, the need for two or more antihypertensive agents often indicates more severe or resistant hypertension, suggesting underlying vascular disease that increases susceptibility to superimposed preeclampsia (9).

Other than diagnostic criteria used, the incidence of chronic hypertension and superimposed preeclampsia depends on a woman’s age, body mass index (BMI), presence of associated medical disorders, and ethnic origin (10, 11). Studies on the population in Southeast Asia remain scarce.

The primary objective of this study was to determine the incidence of superimposed preeclampsia among pregnant women with chronic hypertension in Northeast Thailand. Characteristics, maternal complications, perinatal outcomes, and associated factors of superimposed preeclampsia were also evaluated.

## 2. Material and Methods

A retrospective multicenter cohort study was conducted at Srinagarind Hospital, Khon Kaen University, and Khon Kaen Hospital, a tertiary care facility, Thailand, involving women who were admitted between November 1, 2017, and October 31, 2022. The pregnant women who had been diagnosed with chronic hypertension according to the American College of Obstetricians and Gynecologists (ACOG) guidelines (1) were identified and their medical records were reviewed. Pregnant women with incomplete or lost data and fetal abnormalities were excluded from this study.

Data were collected and reviewed from medical records to confirm the diagnosis. Data collected included baseline characteristics, obstetric data, history of previous preeclampsia, diagnosis, antihypertensive treatment, treatment with aspirin (ASA), MAP during pregnancy, labor and delivery data, and neonatal outcomes. According to available documentation, most women started ASA prophylaxis between 12 and 20 gestational weeks. However, adherence throughout pregnancy could not be reliably determined due to incomplete recording, and therefore, compliance could not be assessed. Cases with missing critical variables were excluded during screening, and among included participants, missingness for key variables was < 5%; therefore, no imputation was performed. The incidence of superimposed preeclampsia was determined. Various characteristics regarding maternal complications, perinatal outcomes, and associated factors were compared between chronic hypertension women with superimposed preeclampsia and those without.

According to ACOG guidelines (1, 12), chronic hypertension in pregnancy is defined as hypertension, SBP ≥ 140 mmHg, DBP ≥ 90 mmHg, or both with at least two determinations at least 4 h apart, diagnosed before pregnancy or before 20 weeks of gestation and hypertension during pregnancy that does not resolve within 12 weeks postpartum.

Superimposed preeclampsia is defined as chronic hypertension complicated with sudden increase in baseline hypertension or a sudden increase in proteinuria, above the threshold for normal (≥300 mg per 24‐h urine collection, protein/creatinine ratio ≥ 0.3, or dipstick reading of 2+) or a clear change from baseline. In the absence of proteinuria, a sudden increase in BP with new onset of any of the following: (1) severe hypertension (SBP ≥ 160 mmHg or DBP ≥ 110 mmHg), (2) thrombocytopenia (platelet count < 100,000/*μ*L), (3) impaired liver function (elevated blood concentrations of liver enzymes to more than twice the upper limit normal concentrations), (4) renal insufficiency (serum creatinine concentration > 1.1 mg/dL or doubling of the serum creatinine concentration in the absence of other renal disease), (5) pulmonary edema, and (6) new‐onset headache and cerebral or visual symptoms. MAP during pregnancy was calculated using the formula MAP = [SBP + 2(DBP)]/3 and was averaged across all antenatal visits between 20 and 28 weeks of gestation. Statistical analysis was performed using STATA Version 10.0 (StataCorp LP, College Station, Texas, United States). A precision‐based sample size estimation was conducted using an expected incidence of 40%, a desired precision of ±5%, and a 95% confidence level, yielding a minimum required sample size of 369 participants. The final sample of 406 women exceeded this requirement and provided adequate statistical precision for the primary outcome.

Descriptive statistics, including number, percentage, mean with standard deviation (SD), or median with interquartile range (IQR), were used to describe various characteristics as appropriate. The incidence of superimposed preeclampsia was reported as a percentage along with a 95% confidence interval (CI). Chi‐square test and Mann–Whitney *U* test were used to compare various characteristics between women with superimposed preeclampsia versus those without. Logistic regression analysis was used to determine the factors associated with superimposed preeclampsia. An adjusted odds ratio (OR) with a 95% CI was estimated. Multicollinearity among predictors was assessed using variance inflation factors (VIFs), all < 2, indicating no significant collinearity. A sensitivity analysis excluding multifetal pregnancies demonstrated results consistent with the primary analysis. A *p* value of < 0.05 was considered statistically significant. The study protocol was approved by the Khon Kaen University Ethics Committee for Human Research (HE651580) and the Khon Kaen Hospital Institute Review Board in Human Research (KEMOU66001).

## 3. Results

**Figure 1:**
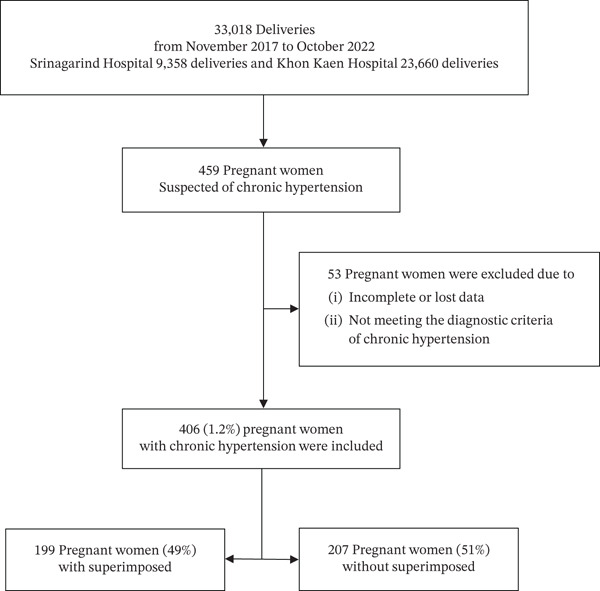
Flow diagram of pregnant women.

There was a total of 33,018 deliveries during the study period. Out of 459 women suspected of having chronic hypertension, 53 were excluded due to incomplete or lost data or not meeting the diagnostic criteria for chronic hypertension. Thus, a total of 406 (1.2%) women with chronic hypertension were identified, as shown in Figure 1.

Superimposed preeclampsia occurred in 199 women, accounting for a rate of 49.0% (95% CI 44.1–53.9). More than half of these women (119, 59.8%) developed superimposed preeclampsia with severe features, while 1 (0.5%) developed eclampsia and 3 (1.5%) developed HELLP syndrome.

Table 1 presents the baseline characteristics of pregnant women with chronic hypertension. Advanced maternal age, defined as 35 years or older, was found in 157 women (38.7%). All the women were of Southeast Asian ethnicity. Multiple pregnancies were documented in 6 women (all of whom were carrying twins). A history of previous preeclampsia was found in 43 women (10.6%). The most common antihypertensive agent used to treat hypertension was methyldopa. Ninety‐four women (23.2%) needed two or more antihypertensive agents to control their BP. Median MAP during GA 20–28 weeks of pregnancy was 103.5 mmHg (IQR 95.7, 109.3) (a MAP of 100 mmHg corresponds to a BP of 135/85 mmHg). In this study, two regimens of ASA were prescribed, with 56.2% received ASA 81 mg/day and 3.9% received 150 mg/day.

**Table 1:** Baseline characteristics of pregnant women with chronic hypertension.

Characteristics	*N* = 406
Age group, *n* (%)	
< 35 years	249 (61.3)
≥ 35 years	157 (38.7)
Race, *n* (%)	
Southeast Asian (Thai)	400 (98.5)
Southeast Asian (Laos, others)	6 (1.5)
BMI category, *n* (%)	
< 18.5 (underweight)	7 (1.7)
18.5–24.9 (normal weight)	112 (27.6)
25–29.9 (preobese)	114 (28.1)
≥ 30 (obesity)	173 (42.6)

Median gestational weight gain (IQR) (kg)	10.5 (7.0, 15.0)
Nulliparous, *n* (%)	115 (28.3)
Multiple pregnancy, *n* (%)	6 (1.5)
In vitro fertilization method, *n* (%)	4 (1.0)
History of previous preeclampsia, *n* (%)	43 (10.6)
Diagnosis of chronic hypertension, *n* (%)	
Before pregnancy	236 (58.1)
During pregnancy	170 (41.9)
Antihypertensive treatment, *n* (%)	
No medical treatment	95 (23.4)
Methyldopa	275 (67.7)
Hydralazine	110 (27.1)

Two or more medication needed, *n* (%)	94 (23.2)
Median MAP during pregnancy (IQR) (mmHg)	103.5 (95.7, 109.3)
ASA prophylaxis, *n* (%)	
ASA 81 mg/day	228 (56.2)
ASA 150 mg/day	16 (3.9)
Underlying medical conditions, *n* (%)	
Gestational diabetes	83 (20.4)
Preexisting and Type I diabetes mellitus	71 (17.5)
Anemia	62 (15.3)


Abbreviations: ASA, aspirin; BMI, body mass index; IQR, interquartile range; MAP, mean arterial pressure.

A comparison of baseline characteristics between pregnant women with and without superimposed preeclampsia is shown in Table 2. Maternal age, BMI, gestational weight gain, parity, onset of chronic hypertension, ASA prophylaxis, and underlying medical conditions were not significantly different between the two groups. A history of previous preeclampsia was more frequently observed in the women with superimposed preeclampsia group (34, 17.1% vs. 9, 4.3%, *p* < 0.001). Median MAP during GA 20–28 weeks of pregnancy was significantly higher in the women with superimposed preeclampsia group (108.7 mmHg [104.7, 114.0]) compared to those without (97.0 mmHg [91.3, 102.7], *p* < 0.001). Additionally, women in the superimposed preeclampsia group tended to require more antihypertensive treatment, with a significant difference observed, especially when two or more antihypertensive agents were needed (69, 34.7% vs. 25, 12.1%, *p* < 0.001).

**Table 2:** Comparison of baseline characteristics between pregnant women with and without superimposed preeclampsia.

Characteristics	Superimposed preeclampsia (*N* = 199)	Without superimposed preeclampsia (*N* = 207)	*p* value
Advanced maternal age (≥ 35 years), *n* (%)	81 (40.7)	76 (36.7)	0.409
BMI category, *n* (%)			0.462
< 18.5 (underweight)	5 (2.5)	2 (1.0)	
18.5–24.9 (normal weight)	58 (29.2)	54 (26.1)	
25–29.9 (preobese)	51 (25.6)	63 (30.4)	
≥ 30 (obesity)	85 (42.7)	88 (42.5)	
Median gestational weight gain (IQR) (kg)	10.5 (7.0, 15.7)	10.4 (7.0, 15.0)	0.620
Nulliparous, *n* (%)	55 (27.6)	60 (29.0)	0.763
History of previous preeclampsia, *n* (%)	34 (17.1)	9 (4.3)	< 0.001
Diagnosis of chronic hypertension, *n* (%)			0.141
Before pregnancy	123 (61.8)	113 (54.6)	
During pregnancy	76 (38.2)	94 (45.4)	
Antihypertensive treatment, *n* (%)			
No medical treatment	37 (18.6)	58 (28.0)	0.025
Methyldopa	150 (75.4)	125 (60.4)	0.001
Hydralazine	70 (35.2)	40 (19.3)	< 0.001
Two or more medication needed, *n* (%)	69 (34.7)	25 (12.1)	< 0.001
Median MAP during pregnancy (IQR) (mmHg)	108.7 (104.7, 114.0)	97.0 (91.3, 102.7)	< 0.001
MAP during pregnancy, *n* (%)			
< 100 mmHg	27 (13.6)	132 (63.8)	< 0.001
≥ 100 mmHg	172 (86.4)	75 (36.2)	
ASA prophylaxis, *n* (%)			0.882
ASA 81 mg/day	111 (55.8)	117 (56.5)	
ASA 150 mg/day	7 (3.5)	9 (4.3)	
Underlying medical conditions, *n* (%)			
Gestational diabetes	42 (21.1)	41 (19.8)	0.746
Preexisting and Type I diabetes mellitus	38 (19.1)	33 (15.9)	0.403
Anemia	34 (17.1)	28 (13.5)	0.320

Abbreviations: ASA, aspirin; BMI, body mass index; IQR, interquartile range; MAP, mean arterial pressure.

Table 3 presents the comparison of delivery and obstetric complications between pregnant women with and without superimposed preeclampsia. The median GA at delivery was significantly lower in the group of women with superimposed preeclampsia (36 weeks [33, 37] vs. 38 weeks [37, 38], *p* < 0.001), with half of these women delivering at less than 37 weeks of gestation. Cesarean section was notably more prevalent in the women with superimposed preeclampsia group, with a total of 145 women (72.9%) undergoing this procedure, out of which 124 (85.5%) were unplanned cesarean sections. Estimated blood loss and nonreassuring fetal status were higher in women with superimposed preeclampsia. However, other obstetric complications, including FGR, oligohydramnios, eclampsia, HELLP syndrome, placental abruption, and admission to the intensive care unit (ICU), did not exhibit significant differences between the two groups.

**Table 3:** Comparison of delivery and obstetric complications between pregnant women with and without superimposed preeclampsia.

Characteristics	Superimposed preeclampsia (*N* = 199)	Without superimposed preeclampsia (*N* = 207)	*p* value
Median GA at delivery (IQR) (weeks)	36 (33, 37)	38 (37, 38)	< 0.001
GA at delivery, *n* (%)			< 0.001
< 28 weeks	12 (6.0)	2 (1.0)	
28–33 weeks	42 (21.1)	6 (2.9)	
34–36 weeks	47 (23.6)	20 (9.7)	
≥ 37 weeks	98 (49.2)	179 (86.5)	

Route of delivery, *n* (%)			< 0.001
Vaginal delivery	52 (26.1)	94 (45.4)	
Operative vaginal deliver	2 (1.0)	7 (3.4)	
Primary cesarean section	94 (47.2)	68 (32.9)	
Repeated cesarean section	51 (25.6)	38 (18.4)	

Median estimated blood loss (IQR) (mL)	300 (200, 400)	200 (110, 400)	< 0.001
Nonreassuring fetal status, *n* (%)	30 (15.1)	14 (6.8)	< 0.001
FGR, *n* (%)	27 (13.6)	16 (7.7)	0.056
Oligohydramnios, *n* (%)	8 (4.0)	11 (5.3)	0.537
Eclampsia, *n* (%)	1 (0.5)	0 (0)	0.490
HELLP syndrome, *n* (%)	3 (1.5)	0 (0)	0.117
Placental abruption, *n* (%)	3 (1.5)	0 (0)	0.117
ICU admission, *n* (%)	7 (3.5)	2 (1.0)	0.100

Abbreviations: FGR, fetal growth restriction; GA, gestational age; HELLP, hemolysis, elevated liver enzyme levels, and low platelet levels; ICU, intensive care unit; IQR, interquartile range.

Table 4 presents the comparison of neonatal outcomes between pregnant women with and without superimposed preeclampsia. The rate of neonatal complications was significantly higher in the group of women with superimposed preeclampsia, including low birth weight (46.5% vs. 20.3%, *p* < 0.001), Apgar score less than 7 at 1 and 5 min (23.5% vs. 7.5%, *p* < 0.001, and 8.0% vs. 0.9%, *p* < 0.001, respectively), and admission to the NICU (33.0% vs. 8.0%, *p* < 0.001). Stillbirth only occurred in the group of women without superimposed preeclampsia, and neonatal death was only found in the group of women with superimposed preeclampsia but with no statistical difference.

**Table 4:** Comparison of neonatal outcomes between pregnant women with and without superimposed preeclampsia (*N* = 412).

Characteristics	Superimposed preeclampsia (*N* = 200)	Without superimposed preeclampsia (*N* = 212)	*p* value
Median birth weight (IQR) (g)	2585 (1770.0, 3192.5)	2990 (2575.0, 3295.0)	< 0.001
Median placental weight (IQR) (g)	600 (500.0, 700.0)	700 (600.0, 800.0)	< 0.001
Low birth weight, *n* (%)	93 (46.5)	43 (20.3)	< 0.001
1‐min Apgar score < 7, *n* (%)	47 (23.5)	16 (7.5)	< 0.001
5‐min Apgar score < 7, *n* (%)	16 (8.0)	2 (0.9)	< 0.001
NICU admission, *n* (%)	66 (33.0)	17 (8.0)	< 0.001
Stillbirth, *n* (%)	0 (0)	2 (0.9)	0.499
Neonatal death, *n* (%)	6 (3.0)	0 (0)	0.013

*Note:* Low birth weight defined as birth weight < 2500g.

Abbreviations: IQR, interquartile range; NICU, neonatal intensive care unit.

Logistic regression analysis was used to determine the factors associated with superimposed preeclampsia, as shown in Table 5. The odds of superimposed preeclampsia were significantly increased in the group of women who had MAP ≥ 100 mmHg during gestational weeks of 20–28 (adjusted OR 9.97, 95% CI 5.95–16.71, *p* < 0.001), needed two or more antihypertensive agents during pregnancy (adjusted OR 2.31, 95% CI 1.30–4.12, *p* = 0.004), and women with a history of previous preeclampsia (adjusted OR 4.52, 95% CI 1.86–10.98, *p* = 0.001).

**Table 5:** Logistic regression analysis of associated factors of superimposed preeclampsia in pregnant women with chronic hypertension.

Variables	Adjusted OR (95% CI)	*p* value
MAP ≥ 100 mmHgduring pregnancy	9.97 (5.95, 16.71)	< 0.001
Combined medication	2.31 (1.30, 4.12)	0.004
History of previous preeclampsia	4.52 (1.86, 10.98)	0.001

Abbreviation: MAP, mean arterial pressure.

## 4. Discussion

The incidence of superimposed preeclampsia in pregnant women with chronic hypertension in the present study was 49.0%, which is higher than previously reported incidences in Thailand (19.0%–44.6%) (13–15), and this trend is expected to continue. Previous studies of the incidence of superimposed preeclampsia globally vary between 20% and 60% (2, 5, 16, 17). The differences in reported incidence may result from variations in populations, diagnostic criteria for superimposed preeclampsia, and management guidelines for chronic hypertension.

Consistent with previous literature, women who developed superimposed preeclampsia in our cohort experienced higher rates of adverse maternal and neonatal outcomes, including preterm delivery, cesarean delivery, increased blood loss, nonreassuring fetal status, low birth weight, low Apgar scores, and NICU admission (18, 19). These findings support the well‐established association between superimposed preeclampsia and adverse perinatal outcomes; however, due to the retrospective design, these relationships should be interpreted as associations rather than causative effects.

The association between superimposed preeclampsia and markers of more severe baseline hypertension is also consistent with previous studies (7, 15, 20). In our analysis, a MAP ≥ 100 mmHg during 20–28 weeks of gestation was strongly associated with an increased likelihood of developing superimposed preeclampsia. Similarly, the need for two or more antihypertensive agents was associated with higher risk, possibly reflecting more severe or less controlled hypertension. These associations align with prior reports suggesting that greater maternal vascular resistance and more intensive antihypertensive requirements may help identify women at higher risk of progression to superimposed preeclampsia.

These findings are consistent with recent global evidence published after 2022, including large, randomized trials and contemporary cohort studies, which emphasize the importance of BP severity and midpregnancy MAP in adverse pregnancy outcomes among women with chronic hypertension. In particular, contemporary data demonstrate a dose–response relationship between higher MAP levels and the risk of preeclampsia, supporting the relevance of MAP as a clinically meaningful marker of risk in current international practice (21, 22).

A history of previous preeclampsia was also associated with superimposed preeclampsia in this cohort. Some earlier studies demonstrated similar associations (7, 10), whereas others did not find a significant relationship (16, 22, 23). Variability in study design, population characteristics, or definitions of chronic hypertension may contribute to these differences. Our findings support the notion that preeclampsia history may serve as an additional clinical marker of susceptibility in pregnant women with chronic hypertension.

In the present study, no significant associations were found between advanced maternal age, obesity, or underlying medical conditions and superimposed preeclampsia, consistent with results reported in previous studies (23). Variation in findings across studies highlights the complex interplay between maternal characteristics and hypertensive disorders of pregnancy. Low‐dose ASA prophylaxis did not demonstrate a statistically significant association with a reduced risk of superimposed preeclampsia in our cohort. Although available records suggested that most women initiated ASA between 12 and 20 weeks of gestation, adherence and duration of use could not be fully evaluated, and the study may have been underpowered to detect differences between dosing regimens. Current guidelines continue to recommend ASA prophylaxis for women at high risk of preeclampsia, including those with chronic hypertension (1, 24, 25), and future prospective studies with standardized ASA protocols and adherence monitoring are warranted.

This study has several strengths, including its multicenter design and large sample size, which enhance the representativeness of the findings within tertiary care settings. However, because both hospitals are referral centers, the incidence of chronic hypertension and superimposed preeclampsia may be higher than in general obstetric populations, and referral bias should be considered. Additionally, the retrospective nature of the study introduces potential limitations, such as incomplete documentation and variations in clinical management. Although missing data were minimal and carefully handled, causal inferences cannot be drawn from this observational design, and the identified relationships should be interpreted as associations rather than causation.

Furthermore, this study was designed to identify factors associated with superimposed preeclampsia rather than to develop or validate a predictive model; therefore, formal model performance measures, such as receiver operating characteristic (ROC) analysis, were not performed. Further prospective studies are warranted to confirm these findings, clarify underlying mechanisms, and evaluate predictive performance.

Overall, this study highlights key clinical factors associated with superimposed preeclampsia in pregnant women with chronic hypertension. Early identification of women at increased risk—particularly those with elevated MAP, multiple antihypertensive requirements, or a history of preeclampsia—may support improved surveillance and timely interventions to optimize maternal and neonatal outcomes.

## 5. Conclusions

Approximately half of the women with chronic hypertension in the present study developed superimposed preeclampsia (49%), higher than previously reported incidences in our country and this trend is expected to continue. MAP ≥ 100 mmHg during pregnancy, the requirement of two or more antihypertensive agents, and a history of previous preeclampsia might be useful tools for predicting superimposed preeclampsia.

## Author Contributions

Conceptualization: A.H. and K.K. Methodology: A.H., K.K., and K.T. Resources: A.H., K.K., R.K., P.S., S.C., C.D., and S.C. Data collection: A.H. Formal analysis: A.H., K.K., and K.T. Data curation: A.H. and K.K. Writing—original draft: A.H. Writing—review and editing: A.H., K.K., R.K., P.S., S.C., C.D., and S.C. Supervision: K.K.

## Funding

No funding was received for this manuscript.

## Disclosure

The paper was presented in the 22nd World Congress in Fetal Medicine, Prague, Czechia (26).

## Conflicts of Interest

The authors declare no conflicts of interest.

## Data Availability

The data that support the findings of this study are available from the corresponding author upon reasonable request.
